# General practitioners' management of mental disorders: A rewarding practice with considerable obstacles

**DOI:** 10.1186/1471-2296-13-19

**Published:** 2012-03-16

**Authors:** Marie-Josée Fleury, Armelle Imboua, Denise Aubé, Lambert Farand, Yves Lambert

**Affiliations:** 1 Department of Psychiatry, McGill University, Montreal, Canada; 2 Douglas Mental Health University Institute Research Centre, Montreal, Canada; 3 Department of Social and Preventive Medicine, Laval University, National Public Health Institute of Quebec, Quebec, Canada; 4 Department of Health Administration, Faculty of Medicine, University of Montreal, Montreal, Canada; 5 Department of Family and Emergency Medicine, Faculty of Medicine, University of Montreal, Montreal, Canada

## Abstract

**Background:**

Primary care improvement is the cornerstone of current reforms. Mental disorders (MDs) are a leading cause of morbidity worldwide and widespread in industrialised countries. MDs are treated mainly in primary care by general practitioners (GPs), even though the latter ability to detect, diagnose, and treat patients with MDs is often considered unsatisfactory. This article examines GPs' management of MDs in an effort to acquire more information regarding means by which GPs deal with MD cases, impact of such cases on their practices, factors that enable or hinder MD management, and patient-management strategies.

**Methods:**

This study employs a mixed-method approach with emphasis on qualitative investigation. Based on a previous survey of 398 GPs in Quebec, Canada, 60 GPs representing a variety of practice settings were selected for further study. A 10-minute-long questionnaire comprising 27 items was administered, and 70-minute-long interviews were conducted. Quantitative (SPSS) and qualitative (NVivo) analyses were performed.

**Results:**

At least 20% of GP visits were MD-related. GPs were comfortable managing common MDs, but not serious MDs. GPs' based their treatment of MDs on pharmacotherapy, support therapy, and psycho-education. They used clinical intuition with few clinical tools, and closely followed their patients with MDs. Practice features (salary or hourly fees payment; psycho-social teams on-site; strong informal networks), and GPs' individual characteristics (continuing medical education; exposure and interest in MDs; traits like empathy) favoured MD management. Collaboration with psychologists and psychiatrists was considered key to good MD management. Limited access to specialists, system fragmentation, and underdeveloped group practice and shared-care models were impediments. MD management was seen as burdensome because it required more time, flexibility, and emotional investment. Strategies exist to reduce the burden (one-problem-per-visit rule; longer time slots). GPs found MD practice rewarding as patients were seen as grateful and more complying with medical recommendations compared to other patients, generally leading to positive outcomes.

**Conclusions:**

To improve MD management, this study highlights the importance of extending multidisciplinary GP practice settings with salary or hourly fee payment; access to psychotherapeutic and psychiatric expertise; and case-discussion training involving local networks of GPs and MD specialists that encourage both knowledge transfer and shared care.

## Background

Mental disorders are a leading cause of morbidity worldwide and widespread in industrialised countries, ranging from 4.3 to 26.4% annually [1,2]. Their substantial burden includes treatment cost, productivity loss, functional impairment, and reduced quality of life. In Canada--as in most of industrialised countries--they rank among the costliest ailments [3]. Mental disorders are mainly treated in primary care, with general practitioners (GPs) providing the initial clinical contact [4]. In the course of a given year, about 80% of the population in industrialised countries consults a GP, of which roughly between 30% and 40% have significant psychological symptoms [5-8]. Given the prevalence of mental disorders, GPs are increasingly called upon to provide appropriate treatment. Psychiatric services alone can neither meet the demand for care nor provide it cost-effectively [9]. GPs also act as brokers, connecting patients with psycho-social services and specialized care providers. This role is crucial as patients in many cases also present substance abuse or physical problems (e.g. diabetes or cardiovascular disease). Compared to specialized care, primary care is considered to be more accessible, less stigmatising, and more comprehensive since it manages physical ailments along with mental disorders [10].

Past reforms in mental healthcare have focused on improving services for the most vulnerable populations (e.g. patients with schizophrenia or bipolar disorder). More recently, under the guidance of the World Health Organization (WHO), countries such as Australia, New Zealand, the United Kingdom (UK), and Canada [11] have attempted to enhance services to individuals with common mental disorders (e.g., depression or anxiety). Efforts have been made to reduce the burden and curb the course of mental disorders through awareness-raising, detection, and rapid access to appropriate treatment. It is reported that more than 75% of patients with depression will relapse or experience recurrence. Lloyd et al. (1996) followed a cohort of patients with mental disorders treated in general practice, and noted that 54% still had specific problems after 11 years, and that 37% had other episodes of illnesses likely associated with chronic mental disorders [12]. These findings confirm the pertinence of close monitoring and long-term approaches to care [13]. Robust primary care has been equated with greater organisational efficiencies and better patient outcomes [14,15]. Accordingly, primary care is the cornerstone of current efforts to improve the performance and results of healthcare systems.

Nonetheless, GPs' ability to detect, diagnose, and adequately treat patients with mental disorders is often considered unsatisfactory. A comparison of research interview results with GPs' detection of mental disorders reveals that 30-70% of GPs' patients go undetected [4]. But longitudinal studies show that only 14% of patients with depression or anxiety remained unrecognised after three years. In national health surveys, from Australia, the UK, the United States, the Netherlands and Canada (39%), it was estimated that only about one third of anxiety and depression patients received treatment [16,17]. Comparing ten high-income countries involved in the WHO Mental Health Survey Initiative [18], estimated minimum adequacy standards range from 18 to 42% among patients receiving treatment for anxiety, mood, and substance disorders. Studies [19-21] usually show that GPs feel comfortable treating most mental disorders, but experience difficulty treating personality disorders, mental disorders associated with substance abuse, and serious disorders (e.g. schizophrenia, bipolar disorder). Other key issues include fragmentation and duplication of services or major gaps in care provision, resulting in preventable emergency-room visits or hospitalisations [22,23]. The literature also mentions lack of integration between primary and specialised care, and inadequate communication among clinicians [24,25].

To help GPs manage patients with mental disorders more effectively, efforts have been made to develop approaches, instruments or guidelines, and collaborative care models, including patient self-management; psychometric diagnostic tools; case management; computerised management systems; continuous training; and shared care for closer coordination among GPs, psychiatrists, and psycho-social professionals [26]. Models of optimal mental healthcare encompass a broad range of management and clinical tools, including step-care and patient-centred approaches.

This article, which is based on previous research on GPs in Quebec [19,27,28], examines GPs' management of mental disorders in an effort to acquire more information regarding means by which GPs deal with mental disorder cases, impact of such cases on their practices, factors that enable or hinder mental disorder management, and patient-management strategies. It also aims to compare GPs' management of common mental disorders and serious mental disorders, and GPs' group profiles (e.g. solo private clinics, private group clinics) as it relates to their involvement with mental disorders. The Quebec healthcare system offers an interesting setting for exploring these topics as it has undergone significant organisational changes, designed to reinforce integrated primary mental healthcare and collaboration among providers. As the development of optimal models of integrated primary care continues to attract worldwide attention, this article contributes to the discussion surrounding service planning for improved mental disorders management, and especially in the context of Quebec's current on-going reforms. In addition, while a number of studies in the last decade have dealt with GPs' collaboration with mental healthcare professionals, few have used mixed-method--especially qualitative investigation--and studied patterns of collaboration in regards to mental disorders generally (both common and serious mental disorders) or the impact of such cases on their practices [29]. More information is also needed regarding factors that enable or hinder GPs' management of mental disorders, and patient-management strategies, which will help in developing more successful care models [30].

### Quebec/Canada primary care system

In Canada, health care is a provincial jurisdiction, which was regionalised over the past two decades. Under the Canada Health Act (1984), all residents are entitled to free inpatient or outpatient 'medically required' care at the point of delivery. Patients receive treatment at publicly funded facilities or are seen by GPs or private medical specialists in the community who charge their provincial health plan for their services. Drug costs are covered mostly by private insurance plans, although 24% of the population have no such insurance. Quebec is the only province to offer a public drug insurance plan to any citizen not covered by a private plan--43% of its population falls within that category [31]. The ratio of GPs per number of residents in Canada is above the average for OECD member-countries (1.12 GPs per 1,000 inhabitants in 2009) [32]. The province of Quebec has a population of 7.5 million, and 7,199 full-time GPs (1.03 GP per 1,000 inhabitants). The province also has 1,074 psychiatrists in practice (13 psychiatrists per 100,000 inhabitants), and 8,469 psychologists (104 psychologists per 100,000 inhabitants while the national average is 48) [33]. In 2001, approximately 80% of consultations with psychologists were within the private system in Canada, with most of the costs paid out-of-pocket or covered by private insurance [34].

A recent Canadian survey found that 23% of GPs work in solo practice, 51% in group practices, and 24% in multidisciplinary teams [35]. About 40% of GPs work in hospitals, and the same proportion in walk-in clinics [36]. In Canada, GPs are paid mainly through fees for services, but also partly through salary at health and social service centres (HSSCs) or hourly fees in hospitals. Close to 25% of the Quebec population lacks a family physician; accordingly, patient volume at walk-in clinics is high. One study estimated that walk-in clinics were a regular source of care for 60% of patients in some regions of Quebec [37]. In view of theses pitfalls, Quebec reforms, launched in 2005 [38,39], seek to have more GPs working in primary care, group practice and with mixed-payment modes, and to promote patient rostering, psycho-social services, and new collaborative models designed to bolster care access and continuity. Family practice groups (FMG) and networks clinics (NC) have emerged as new models. FMGs involve several GPs working together with nurses responsible for patient screening, follow-up, referral, and registration. NCs are similar to FMGs, except that patients are not registered with GPs, and nurses act mainly as liaison agents. Shared care (coordination between GPs and psychiatrists) has also appeared, but is in the early stages of implementation. In addition, mental healthcare teams have been introduced in HSSCs with the goal of having 20 full-time psycho-social professionals along with two GPs assigned to each team serving a population segment of 100,000 adult patients. At this point, about half of the teams in each HSSC have been formed. Each HSSC mental health team manages a 'single access point' where all referrals, from GPs (also allowing self-referral) to public psycho-social services and specialised mental healthcare (e.g. psychiatrists), are centralized at the network level. Quebec has 95 such HSSC local networks delivering primary care services, which are present in 18 regional healthcare jurisdictions (responsible for specialised care). The HSSC local networks are at the core of the healthcare system, where providers combine primary and specialized care to ensure a complete range of services. Strong community-based agencies (e.g. peer and family self-help groups, crisis centres), private psychotherapy clinics, and a network of residential resources complete the mental primary healthcare system offered in the province. Unfortunately, access to mental healthcare is quite problematic in regard to public psycho-social services (at HSSC) or psychiatric care (psychiatrists in hospital settings). In Quebec, the median waiting period for HSSC psycho-social services after referral by a GP is about 40 days per patient [40]. In the case of psychiatric services, the median wait after referral by a GP is eight weeks for elective referrals and two for urgent cases, while there is a 9.5-week waiting period to receive psychiatric treatments after an appointment with a specialist [41]. Very urgent cases however are seen through emergency services.

## Methods

### Design and study population

This study employs a mixed approach with emphasis on qualitative investigation. It focuses on GPs in five Quebec healthcare regions, including nine HSSC local networks in urban, semi-urban, and rural settings. From a previous research sample and quantitative study of 398 GPs representative of Quebec's GP population [19,27,28], 60 GPs were selected for further investigation, i.e. for a more in-depth examination of GP management of mental disorders in view of the results of the first study. Twelve GPs in each of the five regions were considered. They were selected for their representativeness of a variety of practice settings, including solo or group practices in private clinics, HSSCs, hospitals (acute, psychiatric or long-term), walk-in clinics, family medicine groups (FMGs), and network clinics (NCs). Gender representation within the GP sample was also a factor. The first GPs randomly listed (considering region, practice setting and gender) were asked to participate in the research until completion of the sample. Email, postal letter, fax, and telephone contacts were used to reach GPs. The sample was endorsed by the regional directors of general medicine, the mental healthcare directors in the five target geographical settings, and a research advisory committee consisting of key decision-makers in Quebec. Recruitment occurred from April 2009 to March 2010. Participants signed a consent form approved by a university research ethics board.

### Data collection

A 27-item questionnaire and interview guide were used. Both instruments were tested on three GPs not included in the final sample. The questionnaire--a shorter version of the survey used in the preceding research project [28]--covers four aspects: (1) GPs' socio-demographic profile and practice location; (2) continuing medical education; (3) clinical practice features and profile of patients with mental disorders; and (4) comfort level in managing patients with mental disorders. It included categorical or continuous items, with some five-or ten-point Likert scale questions. It was self-administered and required ten minutes to complete. It was used to compare the sample of the first GPs surveyed (the 398 GPs representative of the full Quebec GP population) with the 60 GPs selected for the qualitative investigation. This short questionnaire also provided complementary information (minimal statistics) in view of improving our understanding of the qualitative investigation (i.e. most of non-statistical data).

Development of the interview guide was based on a literature review of primary care and the preceding research project. The guide included three sections. The first section dealt with clinical practice. GPs were asked about (1) their professional background; (2) what they liked and disliked most about mental disorders management; (3) the clinical tools they used to manage mental disorders; (4) their comfort level in dealing with mental disorders; (5) their skill-development methods; (6) the influence of their workplace on mental disorder management; (7) the impact of mental disorder management on their workload or schedule, and their coping strategies; and (8) the incentives for treating more patients with mental disorders. The second section of the interview guide focused on GPs' relationships with mental healthcare networks, their evaluation of the availability of mental healthcare resources in their territory, and their views on healthcare reform. The third section discussed GPs' need for support and collaboration and their ideal model of practice for treating mental disorders. Interviews (lasting 70 minutes) were conducted by one of the three primary authors (25% face-to-face; the rest by phone). They were recorded and transcribed (respondents' anonymity was respected).

### Data analysis and definition of variables

Quantitative and qualitative analyses were performed. Frequency distribution for categorical variables and mean values for continuous variables were computed with SPSS Statistics 17.0. As for qualitative data, transcripts were read by the three primary authors and subsequently coded using NVivo 8. The codes were derived from the literature on primary care related to the interview guide themes. Transcript analysis also generated new codes. The researchers discussed the coding process to ensure accuracy and refine the interpretation of results. Data analysis also involved the reduction and synthesis of information. Reports integrating both quantitative and qualitative data were produced to summarise pertinent results, which were read and discussed by all researchers. A second qualitative analysis was performed (with NVivo) using data associated with GPs' main practice settings (where GPs' spent most of their work hours--recorded in the questionnaire), namely (1) solo private clinics; (2) private group clinics; (3) HSSCs; and (4) hospitals (including general and psychiatric hospitals). Such qualitative data grouping allowed comparisons of GPs' clinical practice with regard to their main settings. Wherever pertinent, data analysis also compared GP management of patients diagnosed with a common mental disorders (e.g. anxiety, depression) with patients with a serious disorders (e.g., schizophrenia, bipolar disorder) with or without concomitant disorders (physical problems, substance abuse). Patients with common mental disorders differ considerably from patient with serious disorders. The former are generally employed; their problems are often less disabling though they may be recurrent or become chronic and the patient may suffer a relapse. The latter (2-3% of the population) are generally unemployed, and need considerable help in many bio-psycho-social areas on a long term basis (Nelson 2006). As for patients with concomitant disorders, the prognoses are not as positive since these patients are usually reported to be more difficult to treat (greater severity, incompatible treatments, etc.); to require more services (especially emergency); to be hospitalised more often; and to be less likely to abide by prescribed treatment [42].

## Results

### GPs' simple, and socio-demographic and workplace profiles

One hundred twenty-four GPs were approached to participate in the research; 29 were excluded because they had moved or retired or were impossible to reach. Sixty agreed to take part in the research, and 35 refused, for a response rate of 63%. The 60-GP and 398-GP samples were compared on key parameters: age, gender, and fee-for-service income (Table 1)--with no significant difference found. However, the 60-GP sample drew much less income from service fees than Quebec's GP population as a whole. Nevertheless, the validity of our qualitative approach draws less on statistical representation than on the richness and depth of information that we obtained from the subjects, and their detailed explanations of the mechanisms by which different factors influenced their behaviours. Furthermore, our sample size allowed us to reach theoretical saturation.

**Table 1:** Comparison between our 60-GP sample, previous research on a 398-GP sample, and Quebec GP population

	60 GPs (%)	398 GPs (%)	P (*X*^2^)	Quebec GPs* (%)	P (*X*^2^)
**Age categories (years)**			0.350		0.350
<35	1 (1.7)	29 (7.3)		13.7	
35-44	9 (15.0)	112 (28.1)		27.5	
45-54	24 (40.0)	170 (42.7)		35.0	
55-64	22 (36.7)	74 (18.6)		18.3	
65+	4 (6.7)	13 (3.3)		5.5	
**Gender distribution**			0.670		0.322
Male	29 (48.3)	194 (48.7)		55.1	
Female	31 (51.7)	204 (51.3)		44.9	
**Income level from fee for services**	54.8 (37.8)	64.9 (39.8)	0.149	74.0	0.005

*[43]

Of the 60-GP sample, 48% were men and 52% women. Mean age was 52, with nearly two-thirds of the sample (65%) aged 45-59; accordingly, a majority (73%) possessed 20 years of experience or more. Approximately half (47%) earned 67-100% of their income from fees for services. Roughly 75% worked in two to four different settings (average: three). Twenty GPs worked mainly in private group clinics, 11 in both solo private clinics and hospitals, and 18 in HSSCs. Among GPs in private group clinics, 50% were also in family medicine groups (FMGs) and 25% in network clinics. Among GPs in solo private clinics or in HSSCs, 27% also were in FMGs.

### Prevalence and management of mental disorders in GPs' practice

Most GPs reported that at least 20% of visits were by patients with mental disorders, generally common mental disorders. About 15% followed patients with serious mental disorders (one to ten per cent of total patients seen). GPs working principally in solo practice or HSSCs reported seeing the greatest number of patients with mental disorders (both common and serious mental disorders). HSSCs were the only setting where GPs followed patients with serious mental disorders more assiduously (Table 2). A large proportion of patients with mental disorders--specifically 35% of patients with common mental disorders and 52% with serious mental disorders--also presented physical ailments and substance abuse. In these cases, GPs treated both mental disorders and physical ailments. Only 10% of GPs reported treatment of physical conditions exclusively when a mental disorder was also present.

**Table 2:** Prevalence of mental disorders among general practitioners' patients, according to their main workplace (%)

Prevalence (%)	Solo private clinics (%)	Private group clinics (%)	HSSCs (%)	Hospitals (%)	Total
**MD**					
0-33	7 (28.0)	10 (40.0)	4 (16.0)	4 (16.0)	25
34-66	-	9 (39.1)	8 (34.8)	6 (26.1)	23
67-100	4 (33.3)	1 (8.3)	6 (50.0)	1 (8.3)	12
**CMD**					
0-33	7 (16.7)	15 (35.7)	10 (23.8)	10 (23.8)	42
34-66	1 (9.1)	4 (36.4)	6 (54.5)	-	11
67-100	3 (42.8)	1 (14.3)	2 (28.6)	1 (14.3)	7
**SMD**					
0-33	11 (19.6)	20 (35.7)	14 (25.0)	11 (19.6)	56
34-66	-	-	3 (100)	-	3
67-100	-	-	1(100)	-	1

***Index***: HSSCs: health and social service centres; MD: mental disorders (common and serious mental disorders); CMD: common mental disorders; SMD: serious mental disorders

A large majority of GPs reported being very comfortable managing patients with common mental disorders (on a scale of 0-10, 85% evaluated their level of comfort at 8-10--very comfortable; "see Additional file 1: *Examples of verbatim statements*"). GPs' explanations for this finding include frequent exposure to patients presenting common mental disorders; keen interest in this type of disorder; regular continuous medical education; and limited access to psychiatric care (resulting in GPs accepting patients they would otherwise refer to a psychiatrist). Other explanations may be found in personal traits reported by these GPs: listening skills, ability to view matters in perspective, empathy, patience, ability to communicate, and an open mind.

Conversely, most GPs reported difficulties managing patients with serious mental disorders (on a scale of 0-10, a majority scored 5-7 and a large minority, 1-4--somewhat or moderately comfortable). Patients diagnosed with a personality disorder, attention deficit hyperactivity disorder (ADHD), eating disorder, sexual addiction and concomitant substance abuse or mental retardation, were also deemed a challenge. GPs who reported difficulties with such patients worked mainly in solo private clinic. Some GPs admitted that they were comfortable monitoring serious mental disorders when patients were stabilised or when more specialised diagnoses were made. Generally, these GPs had an appropriate background, i.e. they had more exposure to mental disorders, belonged to HSSC mental healthcare teams, had experience in a psychiatric hospital or had received special training. While some patients are difficult to handle, most GPs believe that mental disorders generally can be managed in primary care. Exceptions are patients in crisis or decompensation, who should be seen by a psychiatrist and returned to GPs with appropriate treatment protocols when stabilised. More complex cases (as above) and refractory patients with common mental disorders should be referred to shared-care programs. More difficult cases may also be referred to GPs with experience in providing specialised mental healthcare.

### GPs' clinical practices in mental healthcare: diagnosis and treatment processes

GPs generally used their clinical intuition, experience, and the DSM-IV to detect and diagnose mental disorders. Standardised scales or questionnaires were used infrequently (essentially for specific or complex cases). Occasionally, these instruments helped to convince patients who did not recognise their disorders and usher them into care. Few GPs considered using these tools to monitor mental healthcare outcomes. Overall, they were used more frequently by GPs working at HSSCs. Almost all GPs referred patients to psychiatrists when faced with serious conditions, such as a first schizophrenia diagnosis, likely to exceed their ability to provide effective treatment.

GPs' treatments for common mental disorders were based on the following: pharmacotherapy, support therapy, and psycho-education. They did not generally give prescriptions for serious mental disorders; however, they followed up on psychiatric prescriptions and monitored side effects and physical co-morbidities when patients were stabilised. Some GPs possessed psychotherapy training but, given time constraints, were unable to use it. Consequently, insured patients were referred to psychologists in private practice, whose services were deemed more accessible than those in the public system. Most GPs favoured joint pharmacotherapy and psychotherapy as the most effective treatment for common mental disorders. Patients with a serious mental disorder, major personality disorder or concomitant substance abuse disorder were referred to specialised resources, due to time constraints, onerous healthcare demands, and inadequate treatment skills. GPs made few referrals to community-based agencies, due to unfamiliarity with these resources.

GPs closely followed their patients with mental disorders. In the acute phase, visits were frequent: monthly, bi-weekly or even weekly when patients were in crisis or deemed a suicide risk. Prescribed drug use and side effects were closely monitored. As patients stabilised, intervals between visits grew longer depending on the patients' needs and the GP's availability. On average, GPs saw patients with serious mental disorders without concomitant disorders (SMDs) five times a year and patients with concomitant disorders (SMDCs) nine times a year. The average number of follow-up visits was seven for patients with common mental disorders (CMDs) and eight for patients with concomitant disorders (CMDCs). GPs who worked in hospitals reported the greatest number of patient visits annually. Generally, follow-up visits depended on patient needs or functional impairment; evolution of the illness (first episode, crisis, stabilised or chronic phase); concomitant disorders; and involvement of mental healthcare stakeholders (including social support received by patients). GPs spent 30 minutes on average with patients. Patient visits were reported to be longest in HSSCs (on average 36 minutes) and shortest in solo private clinics (24 minutes).

### GPs' management of mental disorders: enabling and hindering factors

For more than half of GPs (37 out of 60, 61.6%), clinical setting positively influenced the propensity to support patients with mental disorders. The nature of remuneration in HSSCs and hospitals (i.e. salary or hourly fees) allowed GPs to spend more time with their patients. These settings also offered better access to diverse mental healthcare resources, collaboration opportunities with colleagues, comprehensive multidisciplinary assessment, and informal partnerships. Even if all referrals had to be made through the HSSC's single access point, GPs in HSSC and hospital settings were given an advantage by being on-site with public mental healthcare resources, either psycho-social (HSSCs) or specialised (hospitals). Psychosocial professionals were also present at some private group clinics, but on the whole GPs were as isolated in such a setting as in solo private clinics. Generally, HSSCs and hospitals were associated with fewer hindering factors than other settings in the management of patients with mental disorders. At all sites, patient rostering was considered useful in mental disorders management. In rural and remote areas, most GPs assumed full responsibility for cases, as there were few or no other resources.

Overall, key obstacles in GPs' management of patients with mental disorders were as follows: difficulty accessing specialised resources; scarcity of mental healthcare workers; and inadequate support and communication among stakeholders. Another obstacle was the lack of stability with respect to structures and staff in the mental healthcare system. More specifically, direct communication with psychiatrists was extremely difficult in contrast to the ease of access to other medical specialists. Long wait times to see psychiatrists and little communication on estimated appointment intervals were reported. GPs felt helpless and isolated when patients' mental disorders were beyond their ability to treat, and emergency departments were the only solution.

Direct communication with psychologists was also a problem. GPs considered that brief written reports or phone conversations with psychologists on treatment objectives, approaches, and therapy planning at the very least would benefit their patients. Improving collaboration with psychologists would also help in completing insurance forms for patients' sick leave and planning return to work more effectively. Generally, private insurance was found to benefit patients since it eliminated financial stress and allowed treatment to progress as planned. However, major insurance-related irritants were reported: too many long and detailed insurance forms to fill, and too few psychotherapy sessions eligible for reimbursement.

For patients without private insurance, the major impeding factors were a lack of psychologists in the public system (with social workers filling the void); and long wait times for accessing psycho-social professionals. This referral process--involving forms to complete--was found to be inefficient, burdensome and bureaucratic. Information on wait times was not readily available, and service quality was occasionally questioned. GPs at HSSCs were more satisfied with single access points than other GPs. Unless professionals were present in the same building, information sharing and cooperation were spotty or absent. Inadequate communication led to duplication and poor service continuity and integration; for example, patients were hospitalised, discharged or referred to psychiatrists without their GP's knowledge.

### Impact of mental disorders management on GPs' practices

Most GPs appreciated their mental healthcare practice (specifically common mental disorders) and viewed it as a source of satisfaction. They also appreciated the doctor-patient relationship and the opportunity to use holistic approaches in keeping with the dominant bio-psycho-social mental healthcare model. Patients were seen as endearing, grateful, and receptive to treatment and advice as compared with patients presenting physical problems. GPs practices also encompassed patients' relatives, with proffered care and treatment usually resulting in positive outcomes.

Most GPs said that management of mental disorders could be burdensome as it required time, flexibility, and emotional investment. Especially onerous were the prevalence and complexity of cases, particularly patients in crisis situations, refractory disorders, and the presence of concomitant physical or substance abuse problems. Concomitant conditions often defeated treatment for mental disorders. Appointments had to be added to GPs' heavy schedules. Too often, GPs had to cope with these patients alone without critical mental healthcare resources. Patient management, especially for serious mental disorders and personality disorders (narcissistic, histrionic, borderline), was viewed as more taxing: poorer compliance to treatment and appointments, and a disturbing presence in the waiting room. About half of the GPs surveyed mentioned inadequate remuneration and time-consuming mental healthcare duties (managing medical records and insurance forms, ensuring proper use of prescribed drugs, alleviating patient distress, monitoring patient recovery). GPs in solo private clinic were more likely to view mental healthcare management negatively. Seeing patients (especially new ones) in walk-in clinics and coping with time pressures (and crowded waiting rooms) were also stressful.

### Strategies promoted by GPs to improve mental disorders management

GPs advocated various strategies to improve mental disorders management and reduce its burden: (1) see these patients at the beginning or the end of the day; (2) reserve longer time slots for them; (3) when scheduling appointments, alternate between patients based on illness and severity; (4) set new appointments for further evaluation or to treat concomitant problems ("one problem-per-visit rule); (5) reserve time slots for crisis situations; (6) extend workdays or shorten lunch and coffee breaks. Time slots at walk-in clinics were used for crisis situations or regular follow-up. Depending on patient needs, GPs' practice settings were switched (short follow-up in solo private clinics, long screening sessions at HSSCs or hospitals). Bypassing the formal referral process and optimising informal collaborative networks led to improved access to care.

University education was generally deemed insufficient training for providing optimal treatment of mental disorders. Accordingly, over half of GPs reported attending three half-day sessions of continuing medical education on mental disorders in the past 12 months. Training in local network settings was perceived as especially relevant when delivered by psychiatrists, as it linked knowledge transfer with shared care, enabling better communication with psychiatrists. Case discussion with mental healthcare professionals was viewed as beneficial to GPs' skills development. Workshops, conferences, lectures, and specialised articles and books were considered key skill-enhancing resources. As the GPs improved their skills, they pursued training and reading opportunities more assiduously, particularly with regard to specific approaches or more uncommon conditions, including cognitive behavioural therapy, eating disorders, and ADHD. The few management tools utilised (e.g. clinical protocol, screening diagnosis questionnaire) were also expected to be helpful to improve patients' follow-up, especially with the more difficult cases. Few patient-centred approaches were employed. The need to expand shared care--increased involvement of psychiatrists and psychologists as well as other professionals including social workers and nurses for serious mental disorders--was deemed critical. On-site mental healthcare services would be particularly welcomed. Shared care can improve service quality and efficiency. It may allow GPs to increase their caseload, a desirable development given the high number of patients without a family doctor. Finally, GPs wished to be more informed about mental health services availability in their practice environment.

## Discussion

This article is designed to add to the body of knowledge on mental disorder management by GPs, and to provide insights into how the management of primary mental healthcare could be improved. While our study casts new light on GP mental disorder management, it has its limitations. First, it may over-represent GPs who are more keenly interested in mental health, thereby accounting for a larger proportion of GPs paid by salary or hourly fees (rather than fee for service) in our sample compared to GPs in Quebec as a whole. Second, the study focused on Quebec: studies must be conducted in other jurisdictions to establish a basis for comparison. Finally, no data were collected on the adequacy of GPs' treatment of patients with mental disorders, which is considered a major issue [4].

Our findings can be summarised in five factors, which are found to drive GPs' management of mental disorders, discussed in this section: (1) environment (international trends, jurisdictions); (2) macro-organisational features and reforms (formal networking, access to resources, preferred management tools); (3) practice settings (remuneration, internal collaboration, types of clientele); (4) GPs' individual characteristics (training, background, interests, informal networking, confidence treating mental disorders); and (5) patient management profiles (attitudes, illness severity, prognoses).

As already mentioned, there is a strong trend toward reinforcing primary care, which is associated with improved healthcare outcomes [44]. In the past decade, countries and smaller jurisdictions (such as Quebec) have implemented critical reforms designed to strengthen primary care [45]. GPs are the first target of reforms; best practices and new instruments (e.g. key performance indicators) have been introduced, particularly with respect to patients with chronic conditions. It has been shown that GPs play a key role in mental disorder management, especially for common mental disorders, and that mental healthcare workers provide effective complementary services [46]. Patients with mental disorders represent a large proportion of GPs' clientele [47]: the second most important group to seek advice according to a recent study [46]--depression alone being the third reason for seeking help [48]. As seen, there have been a number of efforts to improve primary care in Quebec: family medicine groups (FMGs), network clinics (NCs), and shared care, including the consolidation of HSSC mental health teams, and the introduction of HSSC single access points within each local network.

Our results show that Quebec reforms have not yet significantly improved the mental healthcare system, much to GPs' dissatisfaction regarding such macro-organisational features as networking opportunities and access to resources. However, reforms are still in the early stages. As other studies reveal [49,50], FMGs and NCs have not yielded considerable improvements yet, especially in mental healthcare. In our sample, most FMGs and NCs were solo or private group clinics settings that do not promote more effective mental disorder management. Nurses in those settings played a major role, but, as the literature underscored, they usually reported lacking adequate knowledge and training in mental health, and were more willing to treat serious mental disorder than common mental disorder cases [5,51,52]. Shared care in Quebec is still underdeveloped and, as elsewhere, it has not yet led to the expected improvement in access to psychiatric care [53-55]. HSSC mental healthcare teams and single access points were generally viewed as yet more bureaucracy and failed to streamline GPs' access to psycho-social services. Clinical tools such as standardised scales or clinical protocols, too, were not much deployed. Passive dissemination of guidelines to improve the recognition and management of mental disorders, as well as broad education programs, have generally been found to have minimal positive outcomes [56,57].

As for GPs' practice settings, our study showed that some were more adapted to the management of mental disorders. These settings shared the following features: (1) GPs were paid a salary or hourly fees; (2) longer time slots were available for more comprehensive care; (3) psycho-social mental healthcare teams were located on-site; (4) formal and informal networks were present; and (5) multidisciplinary assessment, meetings, and training were encouraged. These are recurring strategies, promoted to enhance mental disorders treatment [6,58-60]. They are generally present in HSSCs and hospitals, making them more suitable settings for the management of serious mental disorders, and complex cases of common mental disorders. Previous studies [61-63] have pinpointed the key role played by HSSCs (or multidisciplinary settings) in the treatment of complex cases involving both physical and mental illness, which are generally associated with poorer prognoses. Other enabling features, found in our study as in others were as follows: knowing the mental healthcare resources and professionals; formal referral procedures; good communication between practitioners; being informed about patients' treatment following referral; and not having time constraints when trying to respond to patient needs [57,64-66].

Irrespective of main practice settings, GPs' individual characteristics also influenced mental disorders management. Generally, GPs who were more confident or enthusiastic about treating patients with mental disorders (1) had undergone more continuing medical education (CME) in mental healthcare; (2) had greater exposure to this type of patient; (3) had keener interest in mental healthcare; (4) possessed enabling traits (empathy, listening skills); (5) favoured multidisciplinary care approaches; and/or (6) developed mental healthcare practices to fill a void (for example, in rural or remote areas where no psychiatric services were available). Generally, these GPs also relied on better informal networks, thus bypassing long wait times and gaining ready access to the formal mental healthcare network. For Anderson and colleagues [67], general practice experience and private life are key drivers, more influential than academic education and professional literature. Other characteristics such as practice size (for group practice settings), gender, and time devoted to work did not stand out as variables enabling mental disorders management--contrary to what some other research demonstrated [6,64,68].

GPs in our study reported confidence in treating patients with depression or anxiety, in accordance with recent findings [4,69]. Though GPs believed they could manage most cases of mental disorders, they viewed collaboration with psychologists or psychiatrists as central to good management. For common mental disorders, GPs preferred integrated pharmacotherapy and psychotherapy, as promoted by best practices [70,71]. Contrary to one study [72], ours found no evidence of fear among GPs that their use of psychotherapy in patient follow-ups would result in losing a good doctor-patient relationship, and control over treatments, or lead to adverse patient outcomes. For serious mental disorders or refractory disorders, GPs preferred shared care, especially a strong relationship with psychiatrists. Very limited access to psychologists (either private practices where insurance is required or public practices where lengthy wait times prevail) and psychiatrists (who were difficult to contact) was noted. As in most countries, this situation seriously impeded shared care, and has led to a greater emphasis on biomedical solutions, such as pharmacotherapy [66,73-75]. Increased access to psychological treatments, especially brief cognitive behavioural treatment--as an alternative to, or jointly with pharmacotherapy--is part of most recent reforms in countries such as the UK and Australia [9]. Specialised programs such as integrated treatment for substance abuse and serious mental disorders have also been promoted [76]. Neither of these innovations has yet emerged in Quebec on an extensive basis, which is also the case in most countries.

GPs in our study insisted on the importance of the doctor-patient relationship as a key component of mental disorders detection and management. Studies [66,77,78] have demonstrated that a patient-centred style of practice, and listening and communication improve the doctor-patient relationship, by creating trust, encouraging empathy, and helping the patient to disclose psychological problems. GPs also found their mental healthcare practice very rewarding, and patients very grateful, especially when they complied with medical recommendations, leading to positive prognoses. Our results belie previous research [66,70] suggesting that GPs viewed patients with common mental disorders as 'burdens', 'frustrating', and 'not particularly attractive'. Even if GPs appreciated their mental healthcare practices, they did limit the number of patients they took on due to the emotional toll and time-consuming nature of providing such care--hindering factors underlined in most studies [66,68,71,79,80]. In our research, negative attitudes especially occurred when GPs were alone and felt ill-prepared to face complex cases [73]. Certainly, rarer forms of mental disorders or serious mental disorders would be of greater interest to GPs with specialised training than to strictly primary-care physicians, as the literature indicates [19,81]. Furthermore, mental healthcare was viewed as financially unrewarding, and as not offering optimal practice conditions. In spite of these obstacles, GPs followed their patients closely (on a bi-weekly or monthly basis). Studies have uncovered problems with regard to the adequacy of care [70], generally affecting more patients who are not rostered, or reflecting the quality of the GPs' services (too-short visits, technical quality of care).

## Conclusions

Our study highlighted the considerable interest shown by GPs in the management of mental disorders (especially common mental disorders) and treating patients in interdisciplinary settings. GPs in our sample found their practice very rewarding. The prevalence of mental disorders has compelled GPs to invest substantially in mental healthcare. On the whole, they welcomed opportunities to diagnose and treat patients with common mental disorders; however, they also faced a number of impediments, including healthcare system fragmentation; lack of communication, resources, and useful clinical tools; and unsuitable modes of remuneration. In Quebec, as in most other jurisdictions, best practices such as patient self-management, step-care therapy (both of which were essentially not discussed), and shared care are as yet underdeveloped. GPs worked mainly in solo practice, relying on their clinical intuition with little clinical or collaborative support. Psycho-social resources, such as cognitive behavioural therapy, are not sufficiently widespread, which too often compelled GPs to turn to pharmacological solutions as the only affordable option for patients.

In light of current reforms and best-practice recommendations, our study promotes, as a 'step-care approach' to system change, increased access to psychologists and psychiatrists, in an effort to strengthen bio-psycho-social modes of treatment and shared care. Development of a network of GPs in multidisciplinary settings with more specialised knowledge of mental disorders would prove beneficial in the treatment of more complex cases. Specialised resources for the treatment of substance abuse (given the prevalence of concomitant disorders) and greater participation by community-based agencies also represent desirable developments. In addition, rostering of patients and salary-based or hourly-fee compensation should be more promoted. Continuing education and case discussion in local networks with psychiatrists and multidisciplinary resources are also recommended as they favour skill and network development, respectively. Finally, government policy, implementation incentives, and support mechanisms must drive reforms, enabling GPs to play a significant role in the management of mental disorders and bolstering integrated bio-psycho-social approaches.

## Competing interests

The authors declare that they have no competing interests.

## Authors' contributions

MJF designed and monitored the study with assistance from DA and LF. AI collected and analysed the data in collaboration with MJF and DA. All authors, including YL, contributed to the design of the manuscript, and interpretation of the data. MJF, in collaboration with AI, drafted the manuscript. All authors read and approved the final manuscript.

## Pre-publication history

The pre-publication history for this paper can be accessed here:

http://www.biomedcentral.com/1471-2296/13/19/prepub

## Supplementary Material

Additional_file 1 **Mental disorders (MD) management in GPs' practice: examples of some qualitative statements**. Click here for file

## References

[B1] WHO, World Mental Health Survey Consortium Prevalence, severity, and unmet need for treatment of mental disorders in the World Health Organisation Mental Health Surveys JAMA 2004 291 21 2581 2590 15173149 10.1001/jama.291.21.2581

[B2] Mathers C Loncar D Projections of global mortality and burden of disease from 2002 to 2030 PLoS Medicine 2006 3 11 e442 10.1371/journal.pmed.0030442 17132052 PMC1664601

[B3] Lim KL Jacobs P Ohinmaa A Schopflocher D Dewa CS Une nouvelle mesure, fondée sur la population, du fardeau économique de la maladie mentale au Canada Maladies chroniques au Canada 2008 28 3 103 110

[B4] Walters P Tylee A Goldberg D Murray RM Kendler KS McGuffin P Wessely S Castle DJ Psychiatry in Primary Care Essential Psychiatry 2008 4 UK: Cambridge University Press 479 497

[B5] Naji SA Gibb J Hamilton RS Lawton K Palin AN Eagles JM How ready are practice nurses to participate in the identification and management of depressed patients in primary care? Primary Care Mental Health 2004 2 47 54

[B6] Stirling A Wilson P McConnachie A Deprivation, psychological distress, and consultation length in general practice Br J Gen Pract 2001 51 456 460 11407050 PMC1314026

[B7] Rabin S Maoz B Shorer Y Matalon A Balint groups as shared care in the area of mental health in primary medicine Ment Health Fam Med 2009 6 139 143 22477904 PMC2838645

[B8] Watson DE Heppner P Roos NP Reid RJ Katz A Population-based use of mental health services and patterns of delivery among family physicians, 1992 to 2001 Can J Psychiatr 2005 50 7 398 406 16086537

[B9] Mykletun A Knudsen AK Tangen T Overland S General practitioners' opinions on how to improve treatment of mental disorders in primary health care. Interviews with one hundred Norwegian general practitioners BMC Health Serv Res 2010 10 35 20144205 10.1186/1472-6963-10-35 PMC2829552

[B10] Rothman AA Wagner EH Chronic illness management: what is the role of primary care? Ann Intern Med 2003 138 3 256 261 12558376 10.7326/0003-4819-138-3-200302040-00034

[B11] Smith GC From consultation-liaison psychiatry to integrated care for multiple and complex needs Aust NZ J Psychiat 2009 43 1 1 12 10.1080/00048670802534358 19085523

[B12] Lloyd KR Jenkins R Mann A Long-term outcome of patients with neurotic illness in general practice BMJ 1996 313 7048 26 28 10.1136/bmj.313.7048.26 8664767 PMC2351423

[B13] Howell C Marshall C Opolski M Newbury W Management of recurrent depression Aust Fam Physician 2008 37 9 704 708 18797527

[B14] Starfield B Shi L Macinko J Contribution of primary care to health systems and health Milbank quarterly 2005 83 3 457 502 10.1111/j.1468-0009.2005.00409.x 16202000 PMC2690145

[B15] Kringos DS Boerma WGW Hutchinson A van der Zee J Groenewegen PP The breadth of primary care: a systematic literature review of its core dimensions BMC Health Serv Res 2010 10 65 20226084 10.1186/1472-6963-10-65 PMC2848652

[B16] Prins M Verhaak PFM van der Meer K Penninx BWJH Bensing JM Primary care patients with anxiety and depression: need for care from the patient's perspective J Affect Disord 2009 119 163 171 10.1016/j.jad.2009.03.019 19419771

[B17] Government of Canada The human face of mental health and mental illness in Canada 2006 Ottawa: Minister of Public works and Government Services Canada

[B18] Wang PS Aguilar-Gaxiola S Alonso J Angermeyer MC Borges G Bromet EJ Bruffaerts R de Girolama G de Graff R Gureje O Use of mental health services for anxiety, mood, and substance disorders in 17 countries in the WHO world mental health surveys Lancet 2007 370 9590 841 850 10.1016/S0140-6736(07)61414-7 17826169 PMC2847360

[B19] Fleury MJ Bamvita JM Tremblay J Variables associated with general practitioners taking on serious mental disorder patients BMC Fam Pract 2009 10 1 41 10.1186/1471-2296-10-41 19515248 PMC2706225

[B20] Rockman P Salach L Gotlib D Cord M Turner T Shared mental health care. Models for supporting and mentoring family physicians Can Fam Physician 2004 50 3 397 402 15318677 PMC2214558

[B21] Wright MJ Harmon KD Bowman JA Lewin TJ Carr VJ Caring for depressed patients in rural communities: general practitioners' attitudes, needs and relationships with mental health services Aust J Rural Health 2005 13 1 21 27 10.1111/j.1440-1854.2004.00641.x 15720311

[B22] Bachrach LL Psychosocial rehabilitation and psychiatry: what are the boundaries? Can J Psychiatr 1996 41 28 35 10.1177/070674379604100108 8919421

[B23] Provan KG Milward BH A preliminary theory of interorganizational network effectiveness: a comparative study of four community mental health systems Admin Science Quart 1995 40 1 33 10.2307/2393698

[B24] Cunningham PJ Beyond parity: primary care physicians' perspectives on access to mental health care Health Aff 2009 28 3 w490 w501 10.1377/hlthaff.28.3.w490 19366722

[B25] Fleury MJ Integrated service networks: the Quebec case Health Serv Manage Res 2006 19 3 153 165 10.1258/095148406777888080 16848956

[B26] Craven M Bland R Better practices in collaborative mental health care: an analysis of the evidence base Can J Psychiatr 2006 51 6 Suppl 1 7s 72s 16786824

[B27] Fleury MJ Bamvita JM Farand L Aubé D Fournier L Lesage A GP group profiles and involvement in mental health care J Eval Clin Pract 2012 18 2 396 403 10.1111/j.1365-2753.2010.01597.x 21114798

[B28] Fleury MJ Bamvita JM Farand L Tremblay J Variables associated with general practitioners taking on patients with common mental disorders Ment Health Fam Med 2008 5 3 149 160 22477863 PMC2777568

[B29] Engstrom S Foldevi M Borgquist L Is general practice effective? A systematic literature review Scand J Prim Health Care 2001 19 2 131 144 10.1080/028134301750235394 11482415

[B30] Mulvale G Hurley J Insurance coverage and the treatment of mental illness: effect on medication and provider use J Ment Health Policy Econ 2008 11 4 177 199 19096092

[B31] Gagnon M Hébert G Improving Health, Reducing Costs; Costs and Benefits of a Universal Pharmacare Regime in Canada 2010 Ottawa: Canadian Health Coalition, IRIS and CCPA

[B32] OECD StatExtracts Health Care Resources: Physicians by categories http://stats.oecd.org/index.aspx?DataSetCode=HEALTH_STAT&lang=fr

[B33] Institut canadien d'information sur la santé Les soins de santé au Canada 2008 2008 Ottawa (Ontario): ICIS

[B34] Moulding R Grenier J Blashki G Ritchie P Pirkis J Chomienne MH Integrating psychologists into the Canadian health care system: The example of Australia CJPH 2009 100 2 145 147 10.1007/BF03405525 PMC6974088 19839293

[B35] Collège es Médecins de Famille du Canada Soutenir les effectifs futurs en médecine familiale au Canada. En fait-on assez aujourd'hui pour se préparer pour demain? Volume Bulletin 2008 Mississauga: Collège des médecins de famille du Canada

[B36] Collège des Médecins de Famille du Canada Sondage national: auprès des médecins 2004 Ottawa: Collège des médecins de famille du Canada, Association médicale canadienne, Collège royal des médecins et chirurgiens du Canada

[B37] Haggerty J Pineault R Beaulieu MD Brunelle Y Goulet F Rodrigue J Gauthier J Continuité et accessibilité des soins de première ligne au Québec: barrières et facteurs facilitants 2004 Ottawa: Fondation canadienne de la recherche sur les services de santé (FCRSS)

[B38] Ministère de la Santé et des Services sociaux (MSSS) Plan d'action en santé mentale 2005-2010 2005 La force des liens. Québec;

[B39] Ministère de la Santé et des Services sociaux (MSSS) Projet de Loi 83. Loi modifiant la Loi sur les services de santé et les services sociaux et d' autres dispositions législatives 2005 Québec;

[B40] Ministère de la Santé et des Services sociaux (MSSS) Guichet d'accès en SM pour 2009-2010. Données à partir de la banque de données OASIS 2011 Québec: Ministère de la Santé et des Services sociaux

[B41] Esmail N Waiting Your Turn: Hospital Waiting Lists in Canada, 2009 Report 2009 Vancouver, British Columbia: Fraser Institute

[B42] Health Canada Best Practices: Concurrent mental health and substance use disorders 2002 Ottawa, Ontario: Health Canada

[B43] Savard I Rodrigue J Des omnipraticiens à la grandeur du Québec. Évolution des effectifs et des profils de pratique. Données de 1996-1997 à 2005-2006 2007 Québec: Direction de la planification et de la régionalisation--FMOQ

[B44] Starfield B Primary Care in the context of health services systems 2005 Université de Montréal; 1 24

[B45] Jiwani I Fleury MJ Organizing Integrated Care: Perspectives from Canadian provinces of Quebec and Ontario Int J Integrated Care in press

[B46] Lester H Freemantle N Wilson S Sorohan H England E Griffin C Shankar A Cluster randomised controlled trial of effectiveness of primary care mental health workers Br J Gen Prac 2007 57 196 203 PMC2042545 17359606

[B47] Watson D Krueger H Primary Health Care Experiences and Preferences: Research highlights Centre for Health Services and Policy Research 2005 Vancouver (BC): The University of British Columbia

[B48] Ekers D Wilson R Depression case management by practice nurses in primary care: an audit Ment Health Fam Med 2008 5 111 119 22477856 PMC2777566

[B49] Ministère de la Santé et des Services sociaux (MSSS) Évaluation de l'implantation et des effets des premiers groupes de médecine de famille au Québec Rapport de recherche 2009 Québec: Ministère de la Santé et des Services sociaux 142

[B50] Ministère de la Santé et des Services sociaux (MSSS) Sondage auprès des infirmières des Groupes de médecins de famille du Québec 2007-2008 Rapport de recherche 2009 Québec: Ministère de la Santé et des Services sociaux, Direction de l'évaluation et Direction de l'organisation des services de première ligne intégrés 54

[B51] Simons L Lathlean J Kendrick T Community mental health nurses' views of their role in the treatment of people with common mental disorders Prim Care Ment Health 2006 4 121 129

[B52] Rosemann T Joest K Körner T Schaefert R Heiderhoff M Szecsenyi J How can the practice nurse be more involved in the care of the chronically ill? The perspectives of GPs, patients and practice nurses BMC Fam Pract 2006 7 14 1 7 16515692 10.1186/1471-2296-7-14 PMC1475585

[B53] Fitzpatrick NK Walker N Nourmand S Tyrer PJ Barnes TRE Higgitt A Hemingway H Shah S The determinants and effect of shared care on patient outcomes and psychiatric admissions An inner city primary care cohort study Soc Psychiatry Psychiatr Epidemiol 2004 39 154 163 10.1007/s00127-004-0721-0 15052398

[B54] Kisely S Campbell LA Taking consultation-liaison psychiatry into primary care Int J Psychiatry Med 2007 37 4 383 391 10.2190/PM.37.4.c 18441627

[B55] Kates N Mazowita G Lemire F Jayabarathan A Bland R Selby P Isomura T Craven M Gervais M Audet D The evolution of Collaborative Mental Health Care in Canada: a Shared Vision for the Future Can J Psychiatr 2010 56 5 1 10

[B56] Bower P Gilbody S Stepped care in psychological therapies: access, effectiveness and efficiency Br J Psychiatry 2005 186 11 17 10.1192/bjp.186.1.11 15630118

[B57] Brazeau CMLR Rovi S Yick C Johnson MS Collaboration between mental health professionals and family physicians: a survey of New Jersey family physicians Prim Care Companion J Clin Psychiatry 2005 7 1 12 14 10.4088/PCC.v07n0102 15841188 PMC1076445

[B58] Ashworth M Clement S Sandhu J Farley N Ramsay R Davies T Psychiatric referral rates and the influence of on-site mental health workers in general practice Br J Gen Pract 2002 52 39 41 11791814 PMC1314199

[B59] McCall LM Clarke DM Rowley G Does a continuing medical education course in mental health change general practitioner knowledge, attitude and practice and patient outcomes? Primary Care Mental Health 2004 2 13 22

[B60] Nixon D Charles-Jones H Saunders T Tanner D Jackson B Managing mental health in primary care: a partnership approach Prim Care Ment Health 2003 1 81 88

[B61] Oud MJ Schuling J Slooff CJ Meyboom-de Jong B: How do General Practitioners experience providing care for their psychotic patients? BMC Fam Pract 2007 8 37 10.1186/1471-2296-8-37 17598879 PMC1933537

[B62] Geneau R Lehoux P Pineault R Lamarche P Understanding the work of general practitioners: a social science perspective on the context of medical decision making in primary care BMC Fam Pract 2008 9 12 10.1186/1471-2296-9-12 18284700 PMC2263046

[B63] Geneau R Lehoux P Pineault R Lamarche PA Primary care practice a la carte among GPs: using organizational diversity to increase job satisfaction Fam Pract 2007 24 2 138 144 10.1093/fampra/cml073 17264070

[B64] Greenaway R Fortune L General practitioners' view of psychological services: a comparison of general practitioners who refer to onsite and offsite services Primary Care Mental Health 2006 4 245 254

[B65] Solano L Pirrotta E Ingravalle V Fayella P The family physician and the psychologist in the office together: a response to fragmentation Ment Health Fam Med 2009 6 2 91 98 22477897 PMC2777604

[B66] Barley EA Murray J Walters P Tylee A Managing depression in primary care: a meta-synthesis of qualitative and quantitative research from the UK to identify barriers and facilitators BMC Fam Pract 2011 12 47 10.1186/1471-2296-12-47 21658214 PMC3135545

[B67] Anderson SJ Troein M Lindberg G General practitioners' conceptions about treatment of depression and factors that may influence their practice in this area. A postal survey BMC Fam Pract 2005 6 1 21 10.1186/1471-2296-6-21 15904500 PMC1180707

[B68] Zantigue EM Verhaak PFM Kerssens JJ Bensing JM The workload of GPs: consultations of patients with psychological and somatic problems compared Brit J Gen Pract 2005 55 609 614 16105369 PMC1463219

[B69] Alexander C Fraser J General practitioners' management of patients with mental health conditions: the views of general practitioners working in rural north-western New South Wales Austr J Rural Health 2008 16 6 363 369 10.1111/j.1440-1584.2008.01017.x 19032209

[B70] Upshur C Weinreb L A survey of primary care provider attitudes and behaviors regarding treatment of adult depression: what changes after a collaborative care intervention? Prim Care Companion J Clin Psychiatry 2008 10 3 182 186 10.4088/PCC.v10n0301 18615167 PMC2446486

[B71] van Rijswijk E van Hout H van de Lisdonk E Zitman F van Weel C Barriers in recognising, diagnosing and managing depressive and anxiety disorders as experienced by Family Physicians; a focus group study BMC Fam Pract 2009 10 52 10.1186/1471-2296-10-52 19619278 PMC2734533

[B72] Pierce D Gunn J GPs' use of problem solving therapy for depression: a qualitative study of barriers to and enablers of evidence based care BMC Fam Pract 2006 8 24 17459150 10.1186/1471-2296-8-24 PMC1866236

[B73] Johnston O Kumar S Kendall K Peveler R Gabbay J Kendrick T Qualitative study of depression management in primary care: GP and patient goals, and the value of listening Br J Gen Pract 2007 57 872 879 17976282 10.3399/096016407782318026 PMC2169333

[B74] Manning C Marr J 'Real-life burden of depression' surveys--GP and patient perspectives on treatment and management of recurrent depression Curr Med Res Opin 2003 19 6 526 531 10.1185/030079903125002117 14594525

[B75] Telford R Hutchinson A Jones R Rix S Howe A Obstacles to effective treatment of depression: a general practice perspective Fam Pract 2002 19 1 45 52 10.1093/fampra/19.1.45 11818349

[B76] Mueser KT Noordsy DL Drake RE Fox L Integrated treatment for dual disorders: A guide to effective practice 2003 New York: Guilford Press

[B77] Zantinge EM Verhaak PFM de Bakker DH Kerssens JJ van der Meer K Bensing JM The workload of general practitioners does not affect their awareness of patients' psychological problems Patient Educ Couns 2007 67 93 99 10.1016/j.pec.2007.02.006 17382508

[B78] Van der Pasch M Verhaak PFM Communication in general practice: recognition and treatment of mental illness Patient Educ Couns 1998 33 97 112 10.1016/S0738-3991(97)00057-8 9732651

[B79] McGarry H Hegarty K Johnson C Gunn J Blashki G Managing depression in a changing primary mental healthcare system: comparison of two snapshots of Australian GPs' treatment and referral patterns Ment Health Fam Med 2009 6 75 83 22477895 PMC2777605

[B80] Oladinni O A survey of inner London general practitioners' attitudes towards depression Prim Care Psychiatr 2002 8 3 95 98 10.1185/135525702125001227

[B81] Lester H Tritter JQ Sorohan H Patients' and health professionals' views on primary care for people with serious mental illness: focus group study BMJ 2005 330 7500 1122 1126 10.1136/bmj.38440.418426.8F 15843427 PMC557894

