# Supplementary material for: General practitioners' management of mental disorders: A rewarding practice with considerable obstacles
Source: BMC Fam Pract. 2012 Mar 16;13:19. doi: 10.1186/1471-2296-13-19 (PMC3355055; doi:10.1186/1471-2296-13-19)
Supplement: Additional_file 1 — Mental disorders (MD) management in GPs' practice: examples of some qualitative statements. [file 1471-2296-13-19-S1.DOC]

**Additional file 1 –** Mental disorders (MD) management in GPs’ practice: examples of some qualitative statements

| ***About comfort levels in dealing with MD and issuing a diagnosis***  *“Generally, I feel comfortable with MD cases that I see most often because I’ve developed clinical acumen. Certainly with the most common MD, such as depression, anxiety disorders, generalized anxiety disorders, and social phobia, I feel comfortable enough to treat them.” GP 041 – Private group clinic**  *“Someone who is schizophrenic and had their first psychotic episode, I see (such cases) once every three years. Of course, I’m not comfortable with these patients, because I don’t see such cases often enough.” GP 082 – Solo private clinic*  *“I think it’s more clinical intuition, but also the DSM IV criteria, clinical experience, and the history of what patient tells us that drive the diagnosis.” GP 080 – Private group clinic*  *“I don’t usually use a scale. I have been in practice for 25 years, and at the beginning of my practice, I used the Beck scale for depression to help me; but I use it less and less because I think I have some clinical intuition. I have learned with my years of experience and that allows me to be confident in my diagnosis.” GP 090 – Hospital*  ***About treatment (common and serious mental disorders)***  *“I always insist on prescribing both types of treatment. – for common mental disorders, depression, I strongly recommend psychotherapy and pharmacotherapy*.*”GP 066 – HSSC***  *“Because of time constraints, psychotherapy is unfortunately something more difficult to insert into my schedule. So, surely, you could say that I am first and foremost a pharmacologist.” GP 087 – Solo private clinic*  *“Usually, I don’t initiate drug treatment for patients with serious MD. I re-prescribe treatments that have already been prescribed by a psychiatrist.” GP 065 – HSSC*  *“I have a few cases of drug abuse but I don’t manage such problems, they are dealt with in conjunction with a centre specializing in toxicology.” GP 040 – Hospital*  *“It depends on a patient’s level of stability. The frequency of the follow-up could be monthly, quarterly; it depends on the patients, what their activities, their needs are. I find it quite variable; it depends on the needs of the patient. I don’t have a rule on a minimum or maximum number of follow-up visits.” GP 025 – Private group clinic* |
| --- |
| ***About factors that enable the management of mental disorders for GPs***  *“I work in a HSSC; I think I’m in the right place to support MD patients. My means of remuneration is not fee for service. So I think it promotes more adequate management of MD patients because that clientele requires more time.” GP 063 – HSSC*  *“I am not only in private practice. I work in a psychiatric hospital as well, and that may be why I take better care of my psychiatric patients, I treat their MD, we have team discussions once a week. So obviously, it gives me a lot of experience. You try powerful drugs, something that is more difficult to do in private practice. First, you gain confidence in this kind of medication, and then you gain experience in using them.” GP 064 – Solo private clinic*  *“I practice physical medicine within a psychiatric hospital. I am surrounded, however, by psychiatrists who make decisions and I’m always aware of how such decisions are taken. This encourages me to take on more patients in my private practice.” GP 085 – Hospital*  ***About factors that hinder the management of mental disorders for GPs***  *“Of course, financial compensation is important. I think that it’s not appropriate for treating patients with MD who require more time, but you’re not being remunerated for that extra time. I believe that a special compensation package should be attached to the management of MD.” GP 040 - Hospital*  *“Referrals in psychiatry are difficult, if not impossible. Waiting times are excessively long; telephone consultations for a quick opinion are also not possible.” GP 049 – Solo private clinic*  *“For those without insurance, access to psychotherapy is very difficult. For ten patients in need, only one will be able to receive psychotherapy services in an HSSC or another place that won’t charge a fee.” GP 070 – Solo private clinic* |
| ***About impact of mental disorder management on GPs’ practice***  *“I have the opportunity to establish more intimate interpersonal relationships with MD patients, to enter in their personal world and share their most intimate preoccupations.” GP 001 – HSSC*  *“MD management is rewarding at some point because the people whom you help are usually very grateful. When the treatment works well, it’s certainly rewarding and patients offer you theirs thanks.” GP 044 – Solo private clinic*  *“It takes time to assess MD patients and they should have frequent follow-up. I don’t think you can see someone* *with MD in fifteen minutes. So it takes a lot of your time.” GP 022 – Private group clinic*  *“I have to prolong my workday when I add mental health cases to my schedule.” GP 003 – Hospital*  *“As I said, it may be more in terms of the energy level required for management. When I have a lot of depressed patients or patients in psychiatry, it may be more exhausting at the end of the day.” GP 051 – Private group clinic*  *“One of the main difficulties is often the time required to convince (patients) to comply with or continue their medication, especially patients with substance abuse issues.” GP 047 – Solo private clinic* |
| ***About strategies promoted by GPs to improve mental disorder management***  *“How are we organized? We do what we can. Often, instead of having lunch at noon, you have lunch at 12:45 because you’ve had to add MD cases in between others patients whose appointment has been set for several months. Sometimes, you start a little earlier: instead of starting at nine o’clock, you’ll start at half past eight because you’ve added a patient. That’s how I organize it.” GP 043 – HSSCs*  *“I see my MD cases at the end of the day.” GP003 – Hospital*  *“The training provided by psychiatrists sponsored by the CME*** or pharmaceutical companies is very helpful.” GP 041 – Private group clinic*  *“I’m thinking specifically about lectures on dealing with MD. You might look at books, as well as information posted on the Internet. I also have some DVDs. What I really enjoy is having the opportunity to just chat with a psychiatrist about a specific case. This is truly helpful in building my confidence in the treatment that I’m providing to a patient.”* *GP 091 – Solo private clinic*  *“The most helpful aspects are probably exchanges and discussions with colleagues, clinical experience, and having easy access to communicate with the psychiatrist. It’s not the same when you can have a discussion and receive a written report of the patient’s visit from the psychiatrist. So it’s really important to be in an environment where there is a close collaboration.” GP 069 – Private group clinic*    *“It’s clear that the informal network will sometimes be an easier way because there are waiting lists in the system. If you have contacts, you will try to use these contacts because you are in trouble with a patient, and if you go through official channels it often takes a long time, and there are waiting lists. It’s unfortunate but sometimes you have to act through your informal network.”* *GP 030 - HSSC* |

* General practitioner code and main place of practice

** Health and social service centers

*** Continue medical education
